# Fixed Drug Eruption to Trimethoprim-Sulfamethoxazole and Doxycycline

**DOI:** 10.7759/cureus.14502

**Published:** 2021-04-15

**Authors:** Clara Y Long, Noelle Wong, Ariel Burns

**Affiliations:** 1 Faculty of Medicine, Dalhousie University, Halifax, CAN; 2 Department of Dermatology, Queen Elizabeth II Health Sciences Center, Halifax, CAN

**Keywords:** doxycycline, fixed drug eruption, polysensitivity, trimethoprim-sulfamethoxazole, adverse drug reaction

## Abstract

Fixed drug eruption (FDE) is a common cutaneous drug eruption. We are the first to report a case of polysensitive FDE to both trimethoprim-sulfamethoxazole (TMP-SMX) and doxycycline. Diagnosis of FDE is largely clinical, and it is important to establish a good medication history to identify the causative agent. Treatment depends on avoidance of the implicated drug.

## Introduction

Fixed drug eruption (FDE) is a type of adverse drug reaction manifesting on the skin. Patients with FDE present with the sudden onset of well-demarcated macules or edematous plaques on the skin or mucous membranes, accompanied by itching or burning [1,2]. There may also be vesicles, bullae, or denuded skin [2]. These lesions appear within days after drug administration and often affect the face, genitals, and acral areas [1]. They resolve after a few weeks following discontinuation of the offending medication, although there can be chronic hyperpigmentation [1,2]. It can be difficult to identify the offending medication due to cross-sensitivity and polysensitivity. Cross-sensitivity is well-described and occurs when several chemically related drugs elicit FDE [3]. In contrast, polysensitivity is rare and occurs when chemically unrelated drugs result in FDE [3]. Here, we report a case of polysensitivity to two unrelated antibiotics, trimethoprim-sulfamethoxazole (TMP-SMX) and doxycycline.

## Case presentation

**Figure 1:**
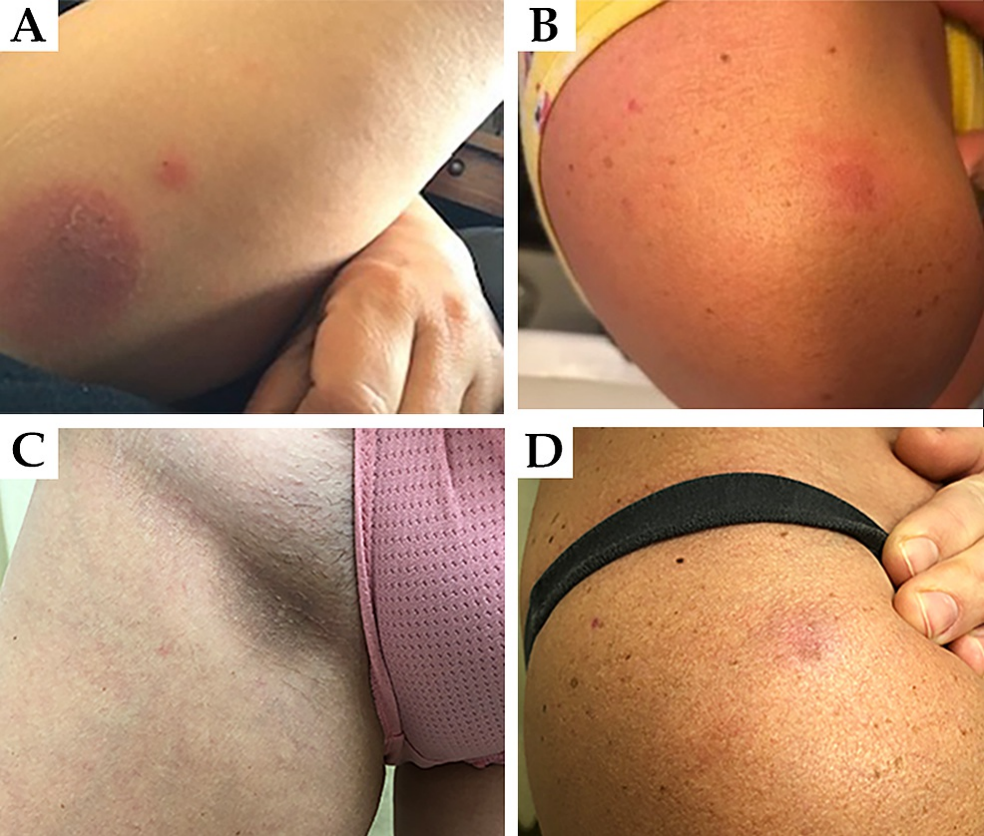
(A) Photograph provided by the patient demonstrating bulla on arm with a gray-violaceous center several days following the first use of TMP-SMX. (B) Photograph provided by the patient demonstrating resolving bulla on the arm several days following the use of doxycycline. (C) Photograph taken at the time of dermatologic consult demonstrating persistent slate-gray hyperpigmentation in the inguinal fold four months following cessation of TMP-SMX. (D) Photograph taken at the time of dermatologic consult demonstrating resolving lesion on the shoulder two weeks following cessation of doxycycline. TMP-SMX, trimethoprim-sulfamethoxazole

A 39-year-old female presented with her third episode of blistering following antibiotic use. Her first episode had occurred two years ago. She described blisters with a gray-purple center that erupted on the hands, arms, legs, and vulva 10-24 hours following the use of TMP-SMX (Figure 1A). These lesions were initially itchy and became sore and tender. This episode lasted two to three weeks before resolving by itself. The second episode occurred two years later, following the use of TMP-SMX. Blisters erupted in the same locations and resolved after two to three weeks. However, they left residual dark gray marks on the skin. Four months later, the third episode occurred 24 hours following the use of doxycycline. These blisters developed on the chest and shoulder and had dusky gray-purple centers that were not as dark as those seen in the previous episodes following TMP-SMX (Figure 1B). While the patient took acetaminophen and ibuprofen for migraines, she reported no issues with either medication. No other medications were used concurrently during these three episodes.

On examination, multiple slate-gray, well-defined patches were seen on the right anterior thigh, right inguinal fold, and left posterior thigh, which were residual areas of post-inflammatory hyperpigmentation following the latest episode with TMP-SMX (Figure 1C). There were areas of fading erythema with a gray hue on the anterior chest, right shoulder, and left flank, which were caused by the most recent episode following doxycycline (Figure 1D). Based on the history and physical examination, the patient was diagnosed with multiple FDE to both TMP-SMX and doxycycline, which was more severe and long-lasting following TMP-SMX. She was advised to avoid these medications to prevent recurrence.

## Discussion

FDE is a delayed-type hypersensitivity reaction mediated by CD8+ T cells [4]. It can be caused by many different drug classes, including non-steroidal anti-inflammatory drugs, acetaminophen, antibiotics, antimalarials, and barbiturates [1]. Among antibiotics, the most common offenders are tetracyclines, sulfamethoxazole, penicillin, and floroquinolone [2,5]. Sulfamethoxazole is responsible for the largest number of FDE worldwide [2].

In this case of polysensitivity, there were two chemically unrelated agents associated with symptom emergence. While both TMP-SMX and doxycycline have been reported as drugs that can cause FDE independently [3,6,7], to our knowledge, this is the first report of polysensitivity to both agents. There has been debate around whether polysensitivity is underreported [8]. However, polysensitivity was reported at one center in 1997 at a rate of less than 0.2% of FDE (one in 600 FDE cases over 20 years) [8]. As the phenomenon is poorly characterized and has been described through sporadic case reports, it is possible that diagnoses have been missed due to a lack of awareness among clinicians. The mechanism is also not well understood. In this case, the lesions caused by doxycycline were markedly different and occurred on different locations than those caused by TMP-SMX, suggesting an antigen-specific mechanism in the pathogenesis of FDE [3]. However, cases of polysensitivity have been reported in which the lesions occurred on identical sites [3]. Additionally, a case has been reported in which FDE resulted when paracetamol-chlormezanone was used in combination but not when these drugs were administered individually, thus potentiating a role for drug interactions in FDE [9]. It is important for clinicians to recognize polysensitivity in order to better characterize the scope of FDE and to appropriately counsel patients when reactions to multiple drugs occur.

The diagnosis of FDE can usually be made clinically based on medication history and physical examination. The hallmark of FDE is reappearance of lesions in the same sites upon re-exposure to the same drug [2]. This was evident in this case following the reuse of TMP-SMX. Important conditions to rule out include erythema multiforme, toxic epidermal necrolysis, and Stevens-Johnson syndrome [10]. While it is important to get an accurate medication history, it can be challenging when there is poor literacy, polypharmacy, and the use of over-the-counter medications [2]. It is also important to consider alternative forms of exposure. This was highlighted by a case report that hypothesized that consumption of antibiotics found in cooked meat caused a woman’s lesions [11]. When there are multiple suspected agents, additional testing can be done through patch testing or with drug challenge [1]. While patch testing is considered safer [10], oral drug challenge is often used to identify antibiotics, given the high rate of false negatives with patch testing due to issues such as low penetrance and low concentration of drug used during patch testing [5]. As prevention is the mainstay for managing FDE, it is crucial to identify the drug causing the reaction. As in this case, patients are advised to avoid these agents or chemically related drugs [2].

## Conclusions

This is the first reported case of FDE polysensitivity to TMP-SMX and doxycycline at independent sites. Because diagnosis of FDE is largely clinical, it is critical to establish a good medication history to identify the causative agent as treatment involves avoiding the implicated drug. Patch testing, oral drug challenge, and skin biopsy can be useful tools when diagnosis is unclear.
